# Amphipathic DNA polymers exhibit antiviral activity against systemic Murine Cytomegalovirus infection

**DOI:** 10.1186/1743-422X-6-214

**Published:** 2009-12-02

**Authors:** Rhonda D Cardin, Fernando J Bravo, Andrea P Sewell, James Cummins, Louis Flamand, Jean-Marc Juteau, David I Bernstein, Andrew Vaillant

**Affiliations:** 1 Division of Infectious Diseases, Cincinnati Children's Hospital Medical Center, University of Cincinnati, Cincinnati Ohio, USA; 2 Southern Research Institute, 431 Aviation Way, Frederick, Maryland, 21701, USA; 3 Rheumatology and Immunology Research Center, CHUQ Research Center and Faculty of Medicine, Laval University, Quebec, Canada; 4 REPLICor Inc 500 Blvd Cartier West, Suite 135, Laval, Quebec, H7V 5B7, Canada

## Abstract

**Background:**

Phosphorothioated oligonucleotides (PS-ONs) have a sequence-independent, broad spectrum antiviral activity as amphipathic polymers (APs) and exhibit potent in vitro antiviral activity against a broad spectrum of herpesviruses: HSV-1, HSV-2, HCMV, VZV, EBV, and HHV-6A/B, and in vivo activity in a murine microbiocide model of genital HSV-2 infection. The activity of these agents against animal cytomegalovirus (CMV) infections in vitro and in vivo was therefore investigated.

**Results:**

In vitro, a 40 mer degenerate AP (REP 9) inhibited both murine CMV (MCMV) and guinea pig CMV (GPCMV) with an IC_50 _of 0.045 μM and 0.16 μM, respectively, and a 40 mer poly C AP (REP 9C) inhibited MCMV with an IC_50 _of 0.05 μM. Addition of REP 9 to plaque assays during the first two hours of infection inhibited 78% of plaque formation whereas addition of REP 9 after 10 hours of infection did not significantly reduce the number of plaques, indicating that REP 9 antiviral activity against MCMV occurs at early times after infection. In a murine model of CMV infection, systemic treatment for 5 days significantly reduced virus replication in the spleens and livers of infected mice compared to saline-treated control mice. REP 9 and REP 9C were administered intraperitoneally for 5 consecutive days at 10 mg/kg, starting 2 days prior to MCMV infection. Splenomegaly was observed in infected mice treated with REP 9 but not in control mice or in REP 9 treated, uninfected mice, consistent with mild CpG-like activity. When REP 9C (which lacks CpG motifs) was compared to REP 9, it exhibited comparable antiviral activity as REP 9 but was not associated with splenomegaly. This suggests that the direct antiviral activity of APs is the predominant therapeutic mechanism *in vivo*. Moreover, REP 9C, which is acid stable, was effective when administered orally in combination with known permeation enhancers.

**Conclusion:**

These studies indicate that APs exhibit potent, well tolerated antiviral activity against CMV infection in vivo and represent a new class of broad spectrum anti-herpetic agents.

## Background

Cytomegalovirus (CMV) is a ubiquitous β-herpesvirus that asymptomatically infects immunocompetent individuals but leads to serious illness and mortality in immunocompromised individuals [1,2]. Currently licensed drugs for the treatment of CMV infection in the United States include Foscarnet, Cidofovir, Ganciclovir and Fomivirisen. These compounds are effective in controlling CMV infection, but emergence of resistance and potentially serious side effects limit their use [2-4]. As such, the need for well-tolerated and potent antiviral compounds with activity against CMV is well recognized.

Amphipathic DNA polymers (APs) are a new class of antiviral compounds based on the sequence-independent activity of phosphorothioated oligonucleotides. The antiviral activity of these compounds against Human Immunodeficiency Virus (HIV-1), Herpes Simplex Virus (and other *herpesviridae*), arenaviruses, and Hepatitis C Virus (HCV) has been previously demonstrated [5-8]. These compounds inhibit both the entry/fusion and attachment of these viruses [5,6,8], and in the case of HIV-1, the amphipathic nature of these compounds was shown to directly mediate their interaction with the core amphipathic α-helices in gp41 [5]. The optimal polymer size (40 mer) for antiviral activity and the specific requirement for phosphorothioation as an enhancer of amphipathic character are well conserved in these unrelated viruses. Moreover, the amphipathic α-helical domains HIV-1 gp41 show a high degree to structural homology to analogous amphipathic domains in the surface glycoproteins of many type 1 fusion viruses [9-15], suggesting that these amphipathic interactions between APs and viral fusion glycoproteins underlie the broad-spectrum antiviral activity of these compounds.

Although the target of AP interaction in viruses other than HIV-1 is not yet known, the structural conservation of amphipathic alpha helical domains in the fusion glycoproteins of most enveloped viruses strongly suggests that the amphipathic domains in surface glycoproteins in *herpesviridae *are a potential target for AP interaction. Thus, the mechanism of action of these compounds appears unrelated to the published mechanism of action for antisense compounds, such as fomvirisen, which is an antisense phosphorothioate oligonucleotide that blocks CMV-specific protein synthesis by hybridizing with mRNA from the major immediate-early transcriptional unit of the CMV genome [16].

APs have been shown to have broad spectrum in vitro antiviral activity against *herpesviridae *including HSV types 1 and 2 (HSV-1, HSV-2), Varicella Zoster Virus (VZV), Human Cytomegalovirus (HCMV), Epstein-Barr Virus (EBV), and Human Herpesvirus 6 types A and B (HHV-6 A/B) [6,17], demonstrating that APs might provide advantages to the currently available therapies to treat herpesvirus infections. Moreover, two amphipathic polymers, REP 9 (a 40 mer degenerate phosphorothioate oligonucleotide) and REP 9C (a 40 mer poly C phosphorothioate oligonucleotide) were shown to be effective topically against HSV-2 infection in a murine model of genital herpes [17], suggesting the potential for antiviral activity in vivo against CMV. Here we report that APs possess anti-CMV activity against two animal CMV strains in vitro and potent, well tolerated prophylactic in vivo efficacy in a murine model of systemic CMV infection via multiple routes of administration and at parenterally administered doses as low as 0.5 mg/kg/day.

## Methods

### Oligonucleotide synthesis

All oligonucleotides for in vitro use were synthesized as described previously [5,7]. Nucleic acid sequences of oligodeoxynucleotides used in this study are as follows:

REP 9 (phosphorothioate) and REP 2015 (phosphodiester): N_40 _(random incorporation of A, G, T and C); REP 9C (phosphorothioate) and REP 2110 (phosphodiester): C_40_; CPG7909: 5' TCGTCGTTTTGTCGTTTTGTCGTT 3' (B-class CPG oligonucleotide, [18]).

Compounds used for in vivo experiments (REP 9, REP 9C) were synthesized under contract with Girindus America Inc. under GMP-like conditions to yield high purity sodium salts.

### Nuclease resistance testing

Oligonucleotide stocks (250 μM in 10 mM Tris, pH 7.2) were diluted to 5 μM in the presence of 1× enzyme reaction buffer. For phosphodiesterase II (Sigma), a working solution of 2 mg/ml was prepared according to the manufacturer's instructions and diluted to 1.3 mg/ml in the reaction buffer containing oligonucleotide. For other enzymes, 100 units (1 μl) of S1 nuclease (Fermentas), or 2 units (2 μl) of Bal 31 (New England Biolabs) or 20 units (1 μl) of exonuclease I (New England Biolabs) were added to the reaction buffer containing oligonucleotide. Reaction conditions were as follows: phosphodiesterase - 24 h at 37°C, S1 nuclease - 4 h at 37°C, Bal31 - 4 h at 30°C, and exonuclease I - 4 h at 37°C. These conditions were established to result in the complete digestion of a 40 mer degenerate phosphodiester oligonucleotide (REP 2015 - see table 1). Controls of all equivalent concentrations of oligonucleotides in the various enzyme buffers were subjected to the same reaction conditions in the absence of enzyme. Following incubation, reactions were stopped by heating at 70-90°C for 4 min before loading onto precast 15% urea-polyacrylamide gels (Biorad). Gels were run for 60 min at 100 volts and a 10 bp ladder (Invitrogen) was used as a size control. Gels were then stained with ethidium bromide (Invitrogen) and oligonucleotides and the degradation products were visualized by UV photography. Stability was assessed relative to no enzyme controls by estimating the proportion of full length oligonucleotide present using a qualitative scale: - = no full length oligonucleotide present, + = 0-25% full length present, ++ = 25-75% full length present, +++ = 75% or greater full length present and ++++ = no degradation of full length oligonucleotide detected. None of these reaction conditions induced any measurable degradation of any of the oligonucleotides in the absence of enzyme (data not shown).

**Table 1:** Nuclease resistance of REP 9, REP 9C and their non-phosphorothioated analogs

			Nuclease resistance (- = fully degraded, ++++ = fully resistant)			
compound	sequence	chemistry	Phosphodiesterase II	S1 Nuclease	Bal 31	Exo I
REP 2015	N_40 _(degenerate)	PO	-	-	-	-
**REP 9**		PS	++	-	++++	++++
						
REP 2110	C_40_	PO	++++	++	-	-
**REP 9C**		PS	++++	++	++++	++++

PO = phosphodiester, PS = phosphorothioate

### Cells and virus

NIH 3T3 cells (ATCC CRL1658) were grown in Dulbecco's modified Eagle's medium (DMEM, Mediatech, Herndon, VA) supplemented with 10% fetal bovine serum (FBS, Hyclone, Thermo-Fisher Scientific), 7.5% Sodium Bicarbonate, 4 mM HEPES, 2 mM L-glutamine, and gentamicin in a humidified 5% CO_2 _incubator at 37°C. Parent stocks of murine cytomegalovirus (MCMV, strain K181+) were kindly provided by Dr. Edward Mocarski (Emory University School of Medicine, Atlanta Georgia). The K181+ virus stocks were grown in NIH 3T3 cells and the resulting tissue culture-passaged stocks were used in all of the in vitro MCMV studies and the majority of in vivo studies. The Smith strain of MCMV was purchased (ATCC, VR-1399) and a salivary gland-passaged virus stock was used in one in vivo study as described in the text. The salivary gland virus stock was prepared from infected salivary glands at 21 days after infection of 6 week old BALB/c mice with the Smith strain [19]. MCMV (Smith) titers were determined by plaque assay on murine embryonic fibroblasts (MEF) from BALB/c mice. MCMV (K181+) titers were determined by plaque assay on NIH 3T3 cells [20,21]. Guinea pig CMV (GPCMV, Strain 22122) was purchased (ATCC, VR-682) and was grown in guinea pig lung fibroblast (GPL) cells (ATCC CCL-158). GPL cells were maintained in F-12 media (Invitrogen Corporation, Grand Island, NY) supplemented with 10% FBS (Hyclone, Thermo-Fisher Scientific) and penicillin/streptomycin in a humidified 5% CO_2 _incubator at 37°C. GPCMV titers were determined by plaque assay on GPL fibroblast monolayers overlaid with media containing 50% Basal Medium Eagle (Sigma-Aldrich Corporation) and 1.5% methylcellulose [22]. All virus stocks were stored at -70°C and re-titered before use in experiments.

### Virological assays

For MCMV plaque assays, dilutions of virus stocks or 10% mouse tissue sonicates were adsorbed onto 70% confluent NIH 3T3 monolayers for one hour at 37°C, then overlaid with 1.5% carboxymethyl cellulose (CMC): 2× modified Eagle's medium (1:1) as previously described [20,21]. After incubation at 5% CO_2 _for 6 days, the CMC overlay was removed and the monolayers were fixed with methanol and stained with Giemsa to determine the number of plaques. To measure the antiviral activity of the compounds against MCMV and GPCMV, plaque reduction assays were performed. For the plaque reduction assay, 100 plaque-forming units (pfu) of MCMV (K181+) or GPCMV were adsorbed onto NIH 3T3 cells or GPL cells and known amounts of compound ranging from 0.01 μM to 1.0 μM were included in the one hour infection of the NIH 3T3 cells (for MCMV) and GPL cells (for GPCMV) and also included in the overlay media of the plaque assay. The assays were performed in duplicate wells in three separate experiments. The IC50 for each compound was calculated as the concentration of compound which reduced the number of plaques by 50% compared to the untreated control. Cytotoxicity of compounds was assessed by Giemsa staining of uninfected murine fibroblasts or Crystal Violet staining of uninfected guinea pig fibroblasts that were incubated with increasing concentrations of REP 9 compound ranging from 1.0 μM to 100 μM and compared microscopically to control monolayers that were not exposed to compound.

### Time-of-Addition assay

To investigate the antiviral activity of REP 9 on MCMV replication when the compound was added at different times during infection, time-of-addition assays were performed using the plaque reduction assay as described above with the following modifications. Briefly, in each well, 10 μM REP 9 or no compound was added with 200 pfu of MCMV (K181+) at the time of infection. After incubation for 2 hours, the virus inoculum was removed and the wells were overlaid with CMC containing 10 μM REP 9 (thus present during entire infection time) or no compound as a control. Additional wells were infected with MCMV for 2 hours or 10 hours in the absence of REP 9, then the virus inoculum was removed, and overlaid with CMC containing 10 μM REP 9, (thus present only at 2 hours or 10 hours after infection). As a control, duplicate sets of wells were set up and cells were washed twice with media to remove excess non-absorbed virus or to remove excess compound. Lastly, similar wells containing 10 μM Ganciclovir (Sigma) added at 2 hours or 10 hours as a control for inhibition of MCMV replication were used. The wells were then incubated at 37°C and 5% CO_2 _for six days and stained with Giemsa to enumerate the average number of plaques in triplicate wells for each inhibition assay. The time-of-addition studies were performed twice and the % reduction of plaques was determined by comparison to the number of plaques in the control wells.

### Mice and infection

Female BALB/c mice were purchased from Jackson Laboratories (Bar Harbor, ME). Three-week old or five-week old mice (8 mice/group) were infected with 1 × 10^5 ^pfu of MCMV (K181+) by intraperitoneal (i.p.) inoculation and maintained under specific pathogen-free conditions at Cincinnati Children's Hospital Medical Center. In one study, 5-7 week old mice were infected with 1 × 10^3 ^pfu of MCMV (Smith) by i.p., intravenous (i.v.), or subcutaneous (s.c.) inoculation and housed in the animal facility at Laval University, Quebec. At various days post infection (dpi), mice were sacrificed, tissues were collected and 10% tissue sonicates were prepared for virus titration by plaque assay.

### In vivo antiviral activity

APs (REP 9, REP 9C), or saline control, were administered to mice by i.p., i.v., or s.c. injection daily at the indicated doses beginning 2 days prior to infection and continued daily for 5 to 6 days as described in the text. For i.p. injection, APs were administered 3 hours prior to infection. On day 0, 3 hours post treatment, the mice were inoculated with virus, and at 3 days post infection (dpi), the mice were sacrificed and the virus titers in the spleen and liver were determined. The spleens were placed in pre-weighed tubes to determine the weight of the spleens at 3 dpi. In some studies, REP 9C was delivered by oral dosing (p.o., 100 μl volume) starting at 2 days prior to infection or in dose response experiments by i.p. injection to 3 week old BALB/c mice starting at 8 days prior to infection. In oral dosing studies, mice were fasted for 3 hours before dosing each day and for 1 hour following dosing. Mice were weighed daily prior to treatment to determine the dose of compound administered to the mice. As a control in some studies, mice were treated daily for 5 consecutive days by i.p. injection of 25 or 50 mg/kg Ganciclovir (GCV, Sigma). In all studies, mice were also monitored for signs of toxicity by weight loss, ruffled fur, and level of activity. For oral REP 9C formulations, the intestinal absorption enhancer n-tetradecyl-β-D-maltopyranoside (TDM, Anatrace, Inc) was prepared at 0.25% in normal saline. The appropriate amount of REP 9C was dissolved in the TDM solution to yield a 400 mg/kg dose per 100 μl solution. A second intestinal absorption enhancer, sodium caprate (C10, Sigma) was formulated in normal saline to yield 100 mg in each oral dose in combination with 400 mg/kg of REP 9C. These solutions were filter sterilized and stored at 4°C and then allowed to come to room temperature prior to administration.

### AP stimulation of cytokine production in human PBMC cells

Human peripheral blood mononuclear cells (PBMCs) were isolated from Leukopaks obtained from Biological Specialty Corporation (Colmar, PA) by centrifugation on a Ficoll-Hypaque density gradient. The cells were washed and resuspended in AIM V (InVitrogen; Carlsbad, CA) serum-free medium (containing 50 μg/ml streptomycin sulfate and 10 μg/ml gentamicin sulfate) at a concentration of 4 × 10^6 ^cells/ml. For cytokine stimulation, the cells were diluted in an equal volume of AIM V medium (containing 2× final drug concentration) to yield a final concentration of 2 × 10^6 ^cells/ml. PBMCs were stimulated in a 48-well format (0.5 ml cells + 0.5 ml drug) for 48 hr with 32 nM of each compound. At study endpoint, cell supernatants were tested in each of the assay platforms to determine the specific cytokines which were stimulated. The following cytokines were measured using kits from Meso Scale Discovery (MSD, Inc., Gaithersburg, MD): Human IFN-γ, IL-1β, IL-2, IL-4, IL-5, IL-6, IL-8 IL-10, IL-12p70, IL-13, TNF-α and IFN Inducible Protein 10 (IP-10). These cytokine levels were determined in culture supernatant by using a multiplex platform developed by MSD. Custom-coated MULTI-SPOT plates (7-, 4-, or 1-Spot 96-well format) were used in which the cytokines could be simultaneously detected within each well of the plate. For each, 10 μl of a calibrator (containing all human cytokine standards) or supernatant was added to appropriate wells of the plate and incubated at room temperature for 1-2 hours with vigorous (300-1000 rpm) shaking. This was followed by three washes with PBS-0.05% Tween-20, addition of 20 μl/well of a 1 μg/ml detection antibody solution (containing antibodies to the cytokines in each kit type), and incubation of the plate at room temperature for 1-2 hours with vigorous shaking. After three washes (PBS-0.05% Tween), 150 μl/well of 2× MSD Read Buffer T was added and the plate was analyzed on a Sector 6000 instrument. Cytokine concentrations in each sample were calculated from calibration curves based on four-parameter logistic algorithms and expressed as pg/ml. In addition, a human IFN-α kit from BioSource (Camarillo, CA) kit was used for this study. The high sensitivity protocol (0-500 pg/ml) was used to determine IFN-α levels in culture supernatants. A volume of 100 μl of calibrator or supernatant was added to appropriate wells of the plate and incubated at room temperature for 1-2 hours. The wells were then washed once in the kit wash buffer, followed by addition of 100 μl of antibody, and incubation of the plate at room temperature for 1-2 hours. After three washes in kit wash buffer, 100 μl of horseradish peroxidase reagent was added, and the plate was incubated at room temperature for 1 hour. After four washes in kit wash buffer, 100 μl of TMB substrate was added, and the plate was incubated for 15 minutes at room temperature. To stop the reaction, 100 μl acid stop solution was added, and the well absorbance were determined at 450 nm. Cytokine concentrations in each sample were calculated from calibration curves based on four-parameter logistic algorithms and expressed as pg/mL.

### Statistical analysis

All virus titer data shown are expressed as mean virus titer (log_10 _pfu/ml) +/- standard error. Statistical analysis was performed using the Student's t test (GraphPad Instat Program). P values of < 0.05 (two-tailed) were considered to indicate a significant difference.

## Results

### APs exhibit in vitro antiviral activity against animal cytomegaloviruses

The strict species specificity of HCMV infection limits the study of HCMV in animal models. To further explore the in vivo antiviral activity of APs against CMV infection, it was first necessary to determine whether REP 9 (a 40 mer degenerate AP) showed antiviral activity in plaque reduction assays against CMV for which small animal models exist. In our assays, REP 9 showed potent activity against both MCMV and GPCMV, with IC50s of 0.045 ± 0.004 μM and 0.16 ± 0.07 μM, respectively, that were comparable to the previously established IC50 of REP 9 against HCMV [17]. In addition to reducing the numbers of plaques observed with increasing concentrations of REP 9, the plaque size also appear to be reduced. A second compound, REP 9C, also showed similar activity against MCMV, with an IC50 of 0.048 ± 0.005 μM. Neither compound demonstrated significant toxicity on fibroblasts, with CC50s > 100 μM. A similar lack of toxicity was reported for REP 9 toxicity on human foreskin fibroblasts (HFFs) [17].

**Figure 1:**
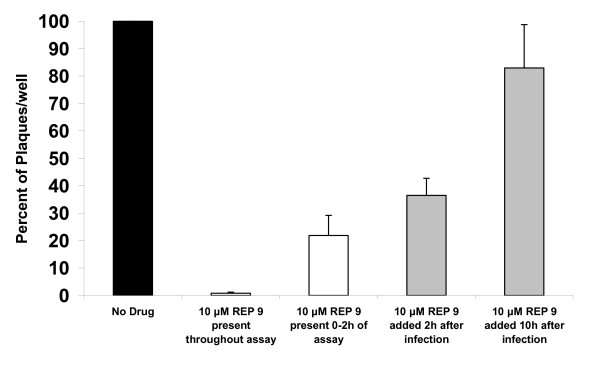
**In vitro REP 9 Activity against MCMV infection at Different Times during Infection**. Plaque reduction assays were performed with no REP 9 compound added or 10 μM REP 9 present throughout the assay, or present for 0-2 hours of the assay, or present only after 2 hours or 10 hours after infection and the remainder of the assay. Addition of 10 μM Ganciclovir in the assay served as a control for inhibition of MCMV replication. Mean antiviral activity is depicted as percentage of plaques in REP 9 wells relative to the percentage of plaques in control wells. Data is from two separate studies and assays performed in triplicate wells.

Previously, it was shown that REP 9 inhibits HSV-2 infection at multiple steps, including binding, entry, and post-entry stages of the virus replication cycle [6]. To determine whether REP 9 acts to inhibit MCMV in a similar fashion, we performed modified time-of-addition assays using MCMV infection of NIH 3T3 cells. Briefly, triplicate wells of cells were infected with MCMV in the absence or presence of 10 μM REP 9 at the beginning of the assay or with addition of REP 9 at 2 hours or 10 hours after infection. As shown in Figure 1, the number of plaques per well were dramatically reduced by 99% compared to control wells when REP 9 was present during the virus inoculation and during the 6 day plaque assay. When the drug was present only during the first two hours of infection, followed by no drug present in the overlay, a 78% reduction in the total number of plaques was observed, indicating that REP 9 inhibits viral infection as early as during the two hours of infection. To further determine whether REP 9 shows activity following post-entry, compound was added after 2 hours post infection or after 10 hours post infection. As shown in Figure 1, addition of REP 9 in the overlay at 2 hours after infection and for the duration of the assay resulted in a 63.5% reduction in the number of plaques observed, whereas the addition of REP 9 in the overlay at 10 hours after infection did not significantly reduce the number of plaques, yielding approximately 83% of the total number of plaques compared to the control wells. Addition of REP 9 at the time of infection or even after 2 hours after infection resulted in markedly smaller plaques detected in the assay, whereas when REP 9 was added at 10 hours after infection, the size of the plaques were only slightly smaller than the size of plaques in the untreated wells. In one study, addition of 10 μM Ganciclovir (GCV) at 2 hours after infection and for the duration of the assay resulted in ~70% reduction in the number of plaques observed (data not shown), as might be expected since the reported IC50 of GCV against MCMV is ~5 μM [23]. As an additional control, wash steps following removal of the virus inoculum and/or drug did not significantly affect the number of plaques observed in the assays (data not shown).

### In vivo effect of REP 9 against murine cytomegalovirus

**Figure 2:**
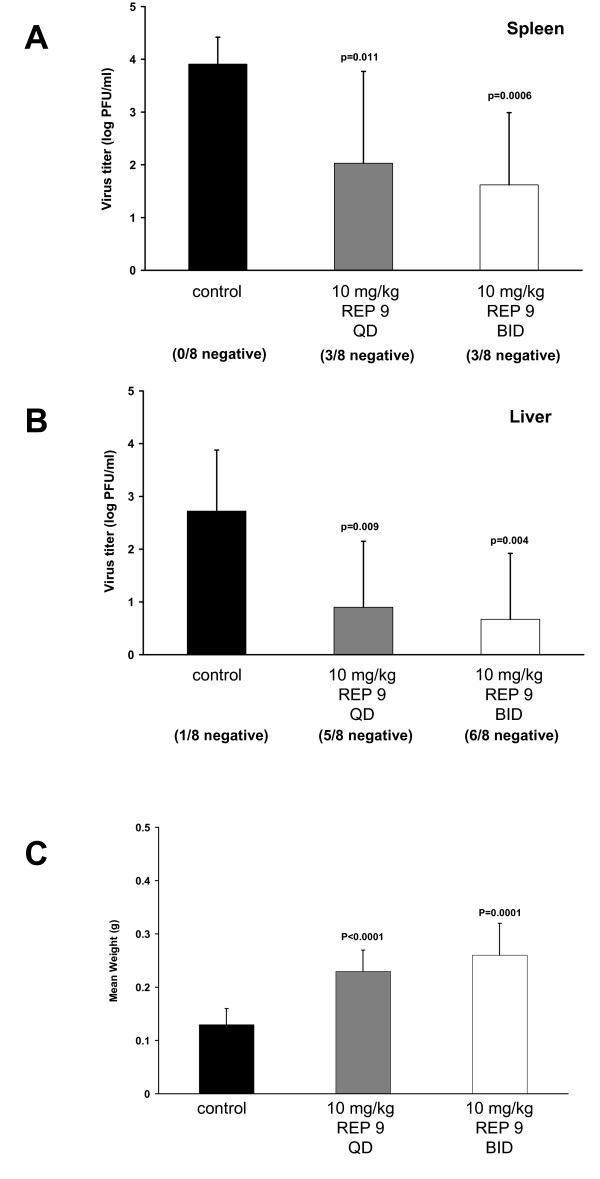
**In vivo Activity of REP 9 against MCMV Replication in the Spleen and Liver**. Virus titer levels of MCMV in spleen (a) and liver (b) and spleen weights (c) of mice treated with 10 mg/kg REP 9 once daily (QD - gray bars) or twice daily (BID - white bars). Five-week old mice (8 mice/group) were treated by daily i.p. administration of saline or REP 9 starting 2 days prior to i.p. inoculation with 1 × 10^5 ^pfu of MCMV (K181+) and continued to 3 days post infection. Data represents results from one typical experiment; Mean +/- standard deviation (n = 8). When observed, animals in each treatment group with no detectable MCMV titer are indicated as x negative/8 total below each bar. The limit of detection for the plaque assay is 10 pfu/ml of tissue homogenate. Virus titers depicted below the limit of detection indicate where samples were negative at the limit of detection but positive for virus in undiluted samples. P values are shown in figure.

Infection of mice with MCMV mimics the infection of humans with HCMV and serves as a well characterized small animal model to assess whether antivirals are active during in vivo MCMV infection. Following i.p. infection, replicating MCMV is found in a number of tissues, including the spleen, liver, lung, and salivary glands [24-28]. Using this model, mice were treated by i.p. injection with REP 9 either once daily (10 mg/kg total) or twice daily (20 mg/kg total) beginning at 2 days prior to i.p. infection with 1 × 10^5 ^pfu of MCMV (K181+). Treatment was continued for 3 days after infection (dpi), at which time the mice were sacrificed and the virus titers in the spleens and livers were determined by plaque assay. As shown in figure 2, REP 9 had a pronounced antiviral effect, significantly reducing MCMV replication in the spleen (figure 2a) regardless of whether the mice were treated with 10 mg/kg REP 9 (p = 0.011) or 20 mg/kg REP 9 (p = 0.0006) as compared to the saline-treated control mice. REP 9 treatment also significantly reduced MCMV replication in the livers (figure 2b) of these mice at both the 10 mg/kg REP 9 (p = 0.009) and 20 mg/kg REP 9 doses (p = 0.004). Moreover, in this experiment, while 8/8 of the spleens and 7/8 of the livers of the saline-treated control mice had detectable virus, fewer mice treated with REP 9 had detectable MCMV in the spleen (5/8 mice at 10 mg/kg and 20 mg/kg) and the liver (3/8 mice at 10 mg/kg and 2/8 mice at 20 mg/kg). Taken together, this data suggests that the antiviral activity of REP 9 may be more potent in the liver than in the spleen. Unexpectedly, REP 9 treatment resulted in splenomegaly as shown by the significant increased spleen weights of the treated mice compared to control mice (figure 2c). Lastly, extending the REP 9 treatment for an additional 2 days to 5 dpi did not significantly reduce the virus levels in the spleens and livers compared to saline-treated control mice (data not shown).

**Figure 3:**
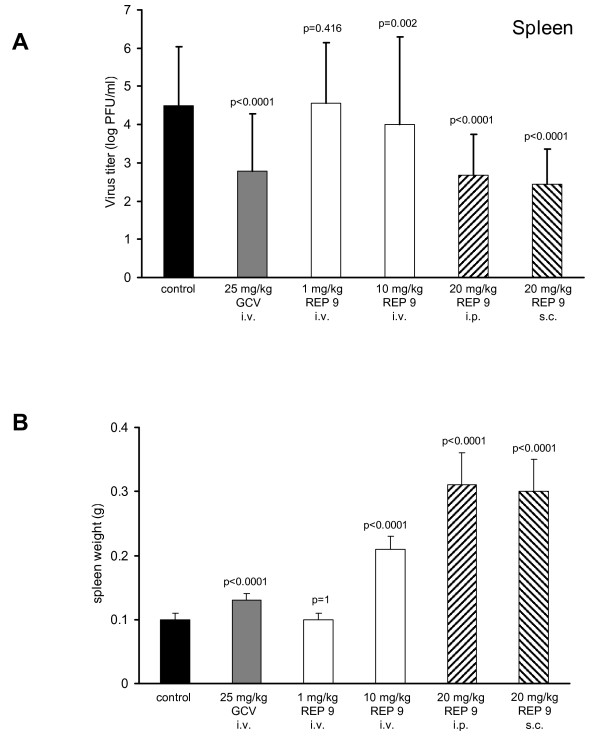
**Antiviral Effects of REP 9 in the Spleen Following Different Routes of Administration**. Viral titers in the spleen (a) and spleen weights (b) of mice treated with either GCV or REP 9 are shown. Five week old mice (8 mice/group) were treated daily with saline or REP 9 starting 2 days prior to i.v. inoculation with 1 × 10^3 ^pfu of MCMV (Smith) and continued until 3 days post infection. REP 9 was administered by i.v, i.p. or s.c. route of injection at 1 mg/kg, 10 mg/kg, or 20 mg/kg. As a control, 25 mg/kg GCV was administered by i.v. route of injection. Data represents results from one experiment; mean +/- standard deviation (n = 8). P values are shown in figure.

Because it is possible that the efficacy of REP 9 therapy was enhanced because treatment and viral infection were both i.p., we next altered both the route of treatment and the route of infection. In this experiment, mice were treated i.v. with 1 or 10 mg REP 9/kg/day or treated i.p. or s.c. with 20 mg REP 9/kg/day, followed by i.v. inoculation with 1 × 10^3 ^pfu MCMV (Smith). Treatment began 2 days prior to i.v. inoculation with MCMV and continued for 3 days after infection. In this study, treatment with REP 9 by either the s.c. or i.p. routes (20 mg/kg/day) or i.v. route (10 mg/kg/day) reduced MCMV replication in the spleen comparable to i.v. treatment with 25 mg/kg/day GCV, indicating that the antiviral activity of REP 9 was independent of the route of REP 9 administration (figure 3a). REP 9 inhibition of virus replication was also not dependent on the MCMV strain or the route of infection. Splenomegaly was also observed at 3 dpi following all three routes of REP 9 administration at 10 mg/kg or 20 mg/kg but was not observed following i.v. administration of 1 mg/kg REP 9 (figure 3b). Mice which were treated i.v. with 25 mg/kg GCV or control mice infected by the i.v. route, did not exhibit splenomegaly. A dose effect was observed following i.v. treatment with 1 mg/kg REP 9 which was not effective at inhibiting virus titers or inducing splenomegaly. In this study, REP 9 (10 mg/kg/day) administered by the i.v. route had a small but significant antiviral effect. However, these mice showed significant signs of distress (inactivity, ruffled fur and weight loss).

### Immunostimulatory activity vs. antiviral activity of APs

**Figure 4:**
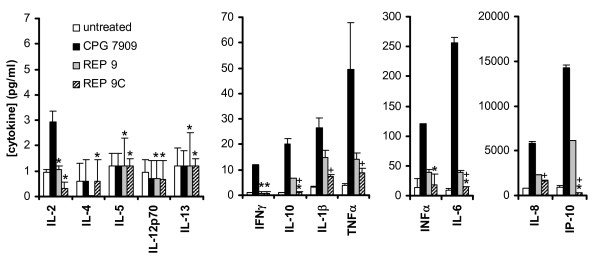
**In vitro Stimulation of cytokine release in human PBMCs by REP 9 and REP 9C**. Human PBMCs were exposed to no compound, CPG 7909, REP 9, and REP 9C, and levels of secreted cytokines after 48 h of induction were determined as described in the material and methods. Data is depicted on different scales (7, 70, 300, and 20,000 pg/ml) to demonstrate differences in cytokine levels measured. Values plotted are mean +/- standard deviation (n = 3). * = statistically insignificant difference in cytokine concentrations compared to untreated controls, + = statistically significant reduction in cytokine concentrations (REP 9C versus REP 9) (p < 0.05).

While REP 9 was active against MCMV infection in all of the initial experiments, REP 9 administration resulted in significant splenomegaly in the treated mice (figures 2c and 3b) compared to saline-treated mice. Due to its degenerate nature, REP 9 contains a small percentage of CpG motifs. The mild CpG-like activity of the REP 9 compound could contribute to the splenomegaly seen in these experiments and also provide antiviral activity. To investigate this issue, we designed an analog of REP 9 which had no CpG motifs, REP 9C (C_40_). To determine whether REP 9C had reduced immunostimulatory activity compared to the REP 9 compound, the ability of REP 9 and REP 9C to stimulate cytokine production in human PBMCs was assessed and compared to a well defined B-class CpG oligo (CPG 7909, [18]) as a control (figure 4). In this experiment, the control compound, CPG7909 at 32 nM exhibited the classical TLR-9 mediated induction of cytokine secretion in human PBMCs, with significant inductions of IFN-γ, IFN-α, IL-1β IL-6, IL-10, IP-10 and TNF-α. At the same concentration, REP 9 stimulated the secretion of these same cytokines as CPG 7909 except IFN-γ but on a much reduced scale, consistent with the presence of mild CpG like activity and observations of splenomegaly in vivo. However, REP 9C exposure to PBMCs resulted in a weaker induction of cytokines in general compared to REP 9. For many cytokines induced by CPG 7909 or REP 9, there was no induction of cytokines in PBMCs by REP 9C. Thus, REP 9C shows minimal or no activity in inducing cytokine secretion in comparison to REP 9 or the positive control CPG 7909.

### In vivo effect of REP 9C against murine cytomegalovirus

**Figure 5:**
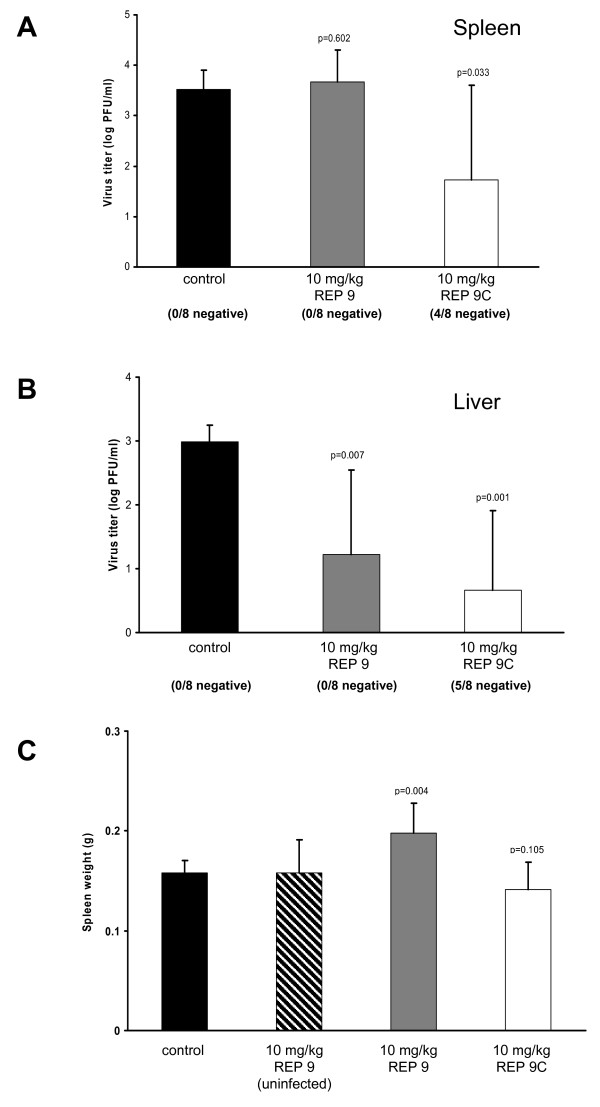
**Comparison of In vivo Antiviral Activity of REP 9 and REP 9C**. Virus titer levels in the spleens (a) and livers (b) and spleen weights (c) are shown for mice treated with 10 mg/kg REP 9 or REP 9C. Five-week old mice (8 mice/group) were treated by daily i.p. administration of saline, REP 9, or REP 9C starting 2 days prior to i.p. inoculation with 1 × 10^5 ^pfu of MCMV (K181+) and continued to 3 days post infection. Data represents results from three separate experiments; Mean +/- standard error (n = 8/experiment). When observed, animals in each treatment group with no detectable MCMV titer are indicated as x negative/8 total below each bar. P values are shown in figure.

REP 9C was then tested in vivo to determine if the lack of CpG motifs and reduced cytokine inducing activity of this analog would still retain the antiviral activity of REP 9 without inducing splenomegaly. Since our previous experience with these compounds indicated that i.p. administration with 10 mg/kg/day was well tolerated, mice were treated once daily with 10 mg/kg of REP 9 or REP 9C beginning at 2 days prior to infection and continuing until 3 dpi. In this study, both REP 9 and REP 9C showed potent antiviral activity in the liver (figure 5b) while REP 9C appeared more efficacious in the spleen (figure 5a). Further, REP 9C did not induce detectable splenomegaly compared to the saline treated control group (figure 5c) while REP 9 treated animals again showed signs of splenomegaly. However, administration of REP 9 to uninfected mice did not induce splenomegaly, suggesting that virus infection contributes to the REP 9-induced splenomegaly observed in our studies.

**Figure 6:**
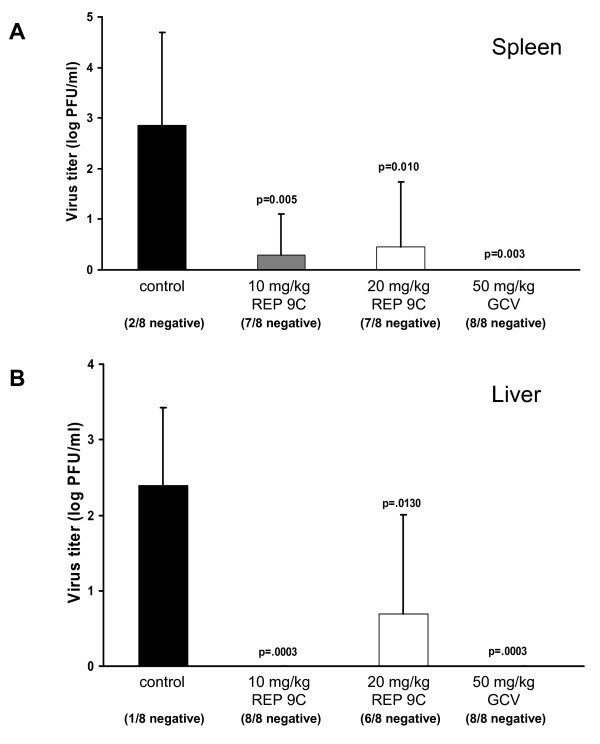
**In vivo Antiviral Comparison of REP 9C and Ganciclovir**. Virus titer levels in the spleens (a) and livers (b) from mice treated with 10 or 20 mg/kg REP 9C or 50 mg/kg Ganciclovir (GCV). Five week old mice (8 mice/group) were treated by daily i.p. administration of saline, REP 9C or GCV starting at 2 days prior to i.p. inoculation with 1 × 10^5 ^pfu of MCMV (K181+) and continued for 3 days. Data represents results from one experiment; mean +/- standard deviation (n = 8). When observed, animals in each treatment group with no detectable CMV titer are indicated as x negative/8 total below each bar. P values are shown in figure.

To further evaluate the antiviral activity of REP 9C in vivo, a second experiment was performed to compare the in vivo antiviral activity of REP 9C with GCV (figure 6). In this experiment, the majority of animals treated with either 10 mg/kg or 20 mg/kg REP 9C as described above had undetectable levels of MCMV in both the spleen (7/8 animals negative at both doses, figure 5a) and liver (8/8 and 6/8 animals negative, respectively, figure 5b). In comparison, 6-7 of the 8 untreated animals had detectable virus in the spleens or livers while none of the animals treated with 50 mg/kg GCV had detectable virus in either organ. Notably, no signs of distress was observed in animals treated with REP 9C at 10 or 20 mg/kg/day, similar to REP 9.

### Nuclease stability of REP 9 and REP 9C

Both REP 9 and REP 9C exhibited comparable antiviral activity in the liver and, to a lesser degree, in the spleen. Since all phosphorothioate oligonucleotides in general are known to accumulate preferentially in the liver and to a lesser extent in the spleen [29-34], the differential activity of these two compounds in the spleen was most likely not due to differences in pharmacokinetic behavior but rather to differences in compound stability. Since nuclease activity is the major degradative pathway of these compounds in vivo, the nuclease stability of both REP 9 and REP 9C were examined (table 1). Both REP 9 and REP 9C and their non-phosphorothioated counterparts were exposed to a variety of nucleases under optimal degradation conditions (see methods) and then subjected to qualitative analysis by denaturing gel electrophoresis. Under our testing conditions, the control, non-phosphorothioated (phosphodiester) degenerate oligonucleotide (REP 2015) had no detectable stability to any of the nucleases tested as expected. REP 9 was fully resistant to Bal31 and Exo 1 digestion but had only moderate stability to Phosphodiesterase II and was not stable to S1 nuclease activity. The poly C phosphodiester oligonucelotide was completely stable to phosphodiesterase digestion but was only moderately stable to S1 nuclease digestion and displayed no resistance to Bal 31 and Exo 1 activity. REP 9C, however, was completely resistant to all enzymes except for S1 nuclease to which it still displayed a high degree of resistance. Thus, REP 9C has a superior nuclease resistance profile to REP 9 and this may account for the better antiviral activity with this analog.

### Antiviral dose response of REP 9C

**Figure 7:**
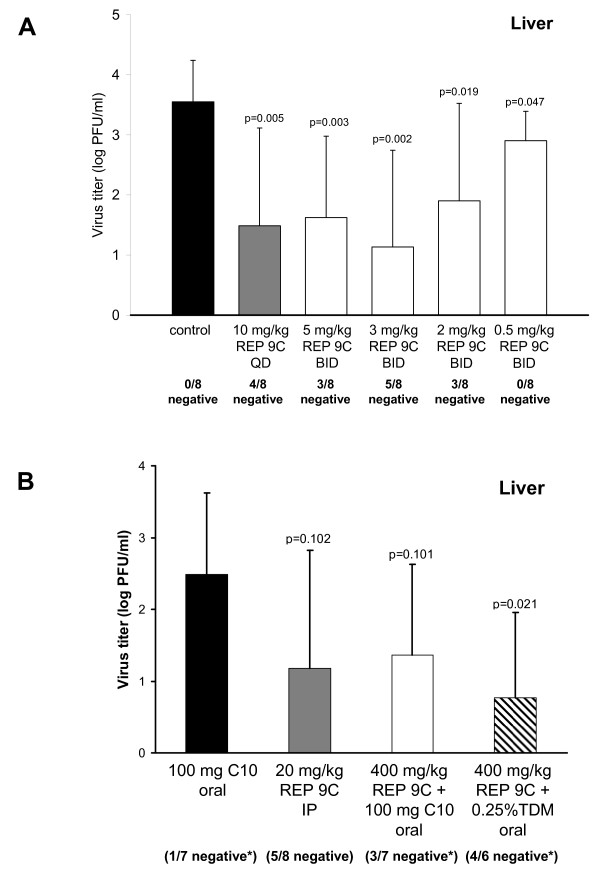
**In vivo Antiviral Dose Effect of REP 9C following Intraperitoneal or Oral Dosing**. (a) Virus titer levels in the livers from mice treated with various doses of REP 9C. Three-week old mice (8 mice/group) were treated by daily i.p. administration of saline or 10 mg/kg (QD), 5 mg/kg (BID), 3 mg/kg (BID), 2 mg/kg (BID), or 0.5 mg/kg (BID) of REP 9C starting at 8 days prior to infection with MCMV and continued for 11 days. Data represents results from one of two separate experiments; Mean +/- standard deviation (n = 8). (b) Virus titer levels in the livers from mice treated orally with 100 mg sodium caprate (C10), 20 mg/kg REP 9C, 400 mg/kg REP 9C in 100 mg C10, and 400 mg/kg REP 9C in 0.25% (w/v) TDM. Five-week old mice (8 mice/group) were treated by daily oral administration of C10 or REP 9C + C10 or TDM starting 2 days prior to i.p. inoculation with 1 × 10^5 ^pfu of MCMV (K181+) and continued to 3 days post infection. Data represents results from one of two separate experiments with similar outcomes; mean +/- standard error (n = 8). The asterisk indicates groups in which mice died due to non-drug related technical difficulties with oral administration. In (a) and (b), mice with no detectable MCMV titer are indicated as x negative/8 total below each bar. P values are shown in figure.

To determine the effect of dose on REP 9C antiviral activity, a dose range study of REP 9C was performed. Initial dose response experiments (figure 3a) indicated that 1 mg/kg/day of REP 9 was ineffective in this model when administered i.v. for 5 days. In this study, the dose of REP 9C ranged from 0.5 mg/kg, twice daily, to 10 mg/kg, once daily, administered i.p. to mice starting at 8 days prior to infection and treated for 11 consecutive days. As shown in Figure 7a, REP 9C administered at 10 mg/kg once daily for 11 consecutive days significantly decreased virus replication in the liver at 3 dpi compared to the saline treated control group (p = 0.005, 4/8 mice MCMV negative). REP 9C also significantly inhibited virus replication in the liver when administered twice daily (BID) at treatment doses of 5 mg/kg (p = 0.003, 3/8 mice MCMV negative), 3 mg/kg (p = 0.002, 5/8 MCMV negative), 2 mg/kg (p = 0.02, 3/8 mice MCMV negative), and 0.5 mg/kg (p = 0.05, 8/8 mice with detectable virus in the liver). Thus, all doses except 0.5 mg/kg BID reduced the numbers of animals with detectable MCMV in their livers. Similar results were seen in a second study (data not shown). It is possible that the known liver accumulation and long half-life of phosphorothioate oligonucleotides in rodent species [29,30] may have lead to increased accumulation of drug, which could explain the increased efficacy seen in this experiment at lower REP 9C doses compared to those using shorter treatments.

### Antiviral activity of REP 9C following oral dosing

The REP 9C compound has been shown to be highly acid resistant [17], making it well suited for oral administration in the absence of a controlled release formulation. To determine whether REP 9C exhibited antiviral efficacy following oral dosing, an oral formulation of 400 mg/kg of REP 9C in 0.25% (w/v) TDM (tetradecyl-β-D-maltopyranoside) or combined with 100 mg of sodium caprate (C10) was used. TDM belongs to a family of alkylated saccharides known to enhance the intestinal absorption of high molecular weight compounds with poor oral bioavailability [35,36] and sodium caprate has previously been shown to substantially enhance to the oral bioavailability of phosphorothioate oligonucleotides in human patients [37,38]. A total volume of 100 μl of either REP 9C + TDM or REP 9C + C10 was delivered orally by gavage with feeding tubes once daily for 5 consecutive days starting at 2 days prior to infection. Control mice were treated with 100 mg C10 in normal saline. Mice were infected i.p. with 1 × 10^5 ^pfu MCMV at day 0 and the livers were collected and titered at 3 dpi. As shown in figure 7b, REP 9C delivered orally with 0.25% TDM or 100 mg C10 significantly reduced the MCMV viral loads in the livers. The reduction in viral titers and number of mice with undetectable MCMV in mice treated with REP 9C + TDM or REP 9C + C10 were comparable to the control group which received 20 mg/kg REP 9C via i.p. injection. Oral administration of REP 9C at 400 mg/kg was shown to have no antiviral activity when administered without an intestinal absorption enhancer (data not shown). Mice treated orally with C10 alone or with C10 in combination with REP 9C developed weight loss, inactivity and ruffled fur but none of these complications were observed in mice which received the TDM + REP 9C oral formulation. The animal deaths which occurred in this study (one mouse in the C10 alone group, one mouse in the REP 9C + C10 group, and two mice in the REP 9C + TDM group) were attributed to mechanical trauma from the oral administration procedure and were not considered to be drug related.

## Discussion

The studies of REP 9 and REP 9C presented here demonstrates for the first time the in vivo activity of APs against systemic MCMV infection in two important tissues, the spleen and liver, where CMV replicates and establishes long term persistent infection. APs have been shown to have antiviral activity in vitro against HSV-1, HSV-2, HCMV, VZV, EBV and HHV-6A/B [6,17], and REP 9 and REP 9C have been previously shown to prevent the topical transmission of HSV-2 [17]. Thus, the in vivo prophylactic activity of REP 9 and REP 9C against MCMV infection in mice suggests that APs may have the potential to be broad spectrum anti-herpetic prophylactic agents in vivo. These initial murine studies presented here are considered an initial proof of concept of the ability of REP 9 and REP 9C to target CMV infection in the liver and spleen.

Traditionally, the activity of phosphorothioate oligonucleotides in rodent models has been difficult to achieve at doses lower than 10 mg/kg/day due to the weaker organ accumulation and much shorter half-life of these compounds compared to primate species [33,34]. Phosphorothioate oligonucleotides accumulate primarily in the liver, kidney, spleen and lung of mammalian species [29-34] and as phosphorothioate oligonucleotides, APs share these same general characteristics (data not shown) and are thus ideally suited to treat viral infections in these organs. Moreover, at equivalent mg/kg dose, phosphorothioate oligonucleotide accumulation in these target organs is known to be substantially increased in primates compared to rodent species while the half life in these target organs increases from 5 days in rodents to 28 days in primates [33,34]. In fact, efficacious dose levels of phosphorothioate oligonucleotides of 10 mg/kg/day in rodent species have translated into effective dosing at 200 mg once weekly in humans [39,40]. This suggests that effective antiviral activity with APs could be achievable with a reasonable dosing regimen in patients.

APs in general have been shown to be entry/attachment inhibitors in HIV-1, HSV-1/2, arenaviruses, and more recently, HCV [5-8]. The inhibition of viral entry/attachment by APs appears to be well conserved in enveloped viruses from divergent families. Similarly, the results from figure 1 indicates that the majority of the REP 9 activity against MCMV appears to occur at early times after infection and thus most likely affects binding and entry as shown for HSV-2 [6]. Some REP 9 activity is also observed when drug is added as late as 10 hours after infection. Thus it seems reasonable to assume that mechanism (s) similar to that reported for REP 9 against HSV-2 are responsible at least in part for the antiviral activity of APs against MCMV infection.

APs have been shown to directly interact with amphipathic alpha helical core fusion domains in HIV-1 [5] which are structurally analogous to the amphipathic alpha-helical structures which are present in gB of HSV [11] and the gH of all herpesviruses [41]. Moreover, these amphipathic domains in gH can be complemented by the core amphipathic alpha-helical domain from HIV gp41 [10]. This heterologous complementation demonstrates the presence of amphipathic alpha-helical structures on the HSV virion surface (as well as all herpesviruses including CMV) with comparable functionality to similar structures in gp41 of HIV-1. Thus, gB and gH may contain domains amenable to AP interaction whose inhibition would be consistent with the entry and attachment inhibition observed with these compounds in HSV-1/2 [6] and with the antiviral activity consistent with entry inhibition in CMV. While the target of AP interaction in CMV has not yet been defined, these observations strongly suggest that interactions of APs with gB and or gH in CMV may be responsible for the inhibition of CMV viral entry.

In murine models, treatment with CpG ODN reduces the severity and time course of infection and facilitates the clearance of viruses such as HSV type 2, Friend retrovirus and influenza [42-44]. Since several studies have shown the involvement of Toll-like receptors (and specifically TLR-9) in the progression of CMV infection [45-48], it is also possible that CpG mediated immunomodulation might be another mechanism whereby REP 9 could indirecty affect viral replication via cytokine stimulation. However, the activity of REP 9C (which is devoid of CpG motifs) in this model system argues that the efficacy of APs in vivo against CMV infection is mainly derived from the direct antiviral activity of APs. Interestingly, REP 9C still maintained residual IL-1β and TNF-α induction in human PBMCs which have pro-viral [49] or antiviral activity [50], respectively. While this cytokine induction in human PBMCs in vitro cannot be directly translated into potential immunomodulatory activity in murine models in vivo, it is possible that some form of cytokine induction may influence the overall antiviral activity of REP 9C in vivo. Splenomegaly was also observed in these studies but only with REP 9 and only in MCMV-infected animals treated with REP 9. It is possible that splenomegaly induced by REP 9 only during MCMV infection could be due to cytokine induction. Further studies are needed to address the underlying mechanism of REP 9-induced splenomegaly.

Phosphorothioate oligonucelotides are known to have limited stability to acid hydrolysis below pH 5, and even when delivered interduodenally to bypass gastric acidity, require intestinal absorption enhancers to achieve systemic exposure [37]. REP 9C is resistant to acid hydrolysis for 24 h at pH1 [17]. This acid resistance is due to the i-plex DNA structure adopted by polypyrimidine sequences [51]. This i-plex structure may also be a factor in the enhanced nuclease stability of REP 9C. The acid stability of REP 9C is predicted to overcome the need for protection from gastric degradation, one of the hurdles in the development of an oral formulation with phosphorothioate oligonucleotides. This is supported by the antiviral activity of an oral formulation of REP 9C with either of two different intestinal absorption enhancers in the absence of any protection from acidic degradation in the digestive tract. The activity of 400 mg/kg of orally administered REP 9C was comparable to that of 20 mg/kg of REP 9C administered parenterally, demonstrating that REP 9C can maintain its bioactivity after passing though the digestive tract. Although the calculated minimum oral bioavailability of REP 9C with these formulations was only 5%, it should be stressed that these experiments were performed with crude formulations which were not optimized for efficient intestinal absorption. Moreover, given the long half-life of these compounds in primates and their preferential accumulation in organs where CMV infection and replication occurs, only modest improvements in oral bioavailability may be necessary to have an oral formulation that would be viable as a prophylactic treatment for CMV in the clinic.

The studies reported here have some limitations. We only evaluated prophylactic administration because CMV infections in rodent systems such as the murine model employed in this study only display transient infections of the spleen and liver, making the therapeutic activity of antiviral compounds difficult to assess. While the use of immunocompromised murine models of CMV infection are also considered useful for evaluating the efficacy of antiviral compounds against CMV infection [52], the efficacy of REP 9 or REP 9C was not further assessed in these models. Investigating the therapeutic activity of APs against CMV infection is the next important step in assessing the clinical potential of these compounds against established CMV infection.

There is a need for antivirals which are effective against multiple human herpesviruses that could be used prophylactically for transplant and severely immunocompromised patients. APs have been shown to have antiviral activity in vitro against HSV-1, HSV-2, HCMV, VZV, EBV, and HHV-6A/6B [6,17]. Importantly, REP 9 and REP 9C appear to be well tolerated and show in vivo activity against HSV-2 [17]. The experiments reported here extend these observations to a systemic infection with another herpesvirus that is an important pathogen in the immunocompromised. Thus, REP 9 and REP 9C may have the potential to be broad-spectrum anti-herpetic prophylactic agents.

## Conclusion

These studies indicate that APs exhibit potent, well tolerated antiviral activity against CMV infection in vivo and represent a new class of anti-herpetic agents.

As a well-tolerated prophylactic agent, APs may be useful to prevent CMV reinfections or reactivations in CMV-positive liver or lung transplant recipients or to prevent primary infection in CMV negative transplant recipients. These compounds may also be useful when combined with currently available drugs for the treatment of CMV. Moreover, since APs are equally effective in vitro against acyclovir and foscarnet resistant herpesviridae [17], these compounds may be useful to treat drug-resistant CMV strains in vivo.

## Competing interests

The authors at Cincinnati Children Hospital Medical Center or Leval University do not have competing interests in the studies presented. The antiviral compounds were supplied by REPLICor, Inc, of which JM and AV are employees.

## Authors' contributions

RC, FB, AS, and LF performed the in vitro virology assays and in vivo studies. RC, AV, and DB drafted the manuscript. AV designed and oversaw manufacture of all oligonucleotides used in the study. AV and JC performed the cytokine stimulation assays. AV performed the nuclease resistance assays. RC, AV, DB, and FL participated in the study design and performed statistical analysis. J-MJ participated in the study design. All authors read and approved the final manuscript.

## References

[B1] Pass RF Cytomegalovirus infection Pediatr Rev 2002 23 163 170 10.1542/pir.23-5-163 11986492

[B2] Biron KK Antiviral drugs for cytomegalovirus diseases Antiviral Res 2006 71 154 163 10.1016/j.antiviral.2006.05.002 16765457

[B3] Boivin G Goyette N Gilbert C Humar A Covington E Clinical impact of ganciclovir-resistant cytomegalovirus infections in solid organ transplant patients Transpl Infect Dis 2005 7 166 170 10.1111/j.1399-3062.2005.00112.x 16390409

[B4] Gilbert C Boivin G Human cytomegalovirus resistance to antiviral drugs Antimicrob Agents Chemother 2005 49 873 883 10.1128/AAC.49.3.873-883.2005 15728878 PMC549271

[B5] Vaillant A Juteau JM Lu H Liu S Lackman-Smith C Ptak R Jiang S Phosphorothioate oligonucleotides inhibit human immunodeficiency virus type 1 fusion by blocking gp41 core formation Antimicrob Agents Chemother 2006 50 1393 1401 10.1128/AAC.50.4.1393-1401.2006 16569857 PMC1426958

[B6] Guzman EM Cheshenko N Shende V Keller MJ Goyette N Juteau JM Boivin G Vaillant A Herold BC Amphipathic DNA polymers are candidate vaginal microbicides and block herpes simplex virus binding, entry and viral gene expression Antivir Ther 2007 12 1147 1156 18240855

[B7] Lee AM Rojek JM Gundersen A Stroher U Juteau JM Vaillant A Kunz S Inhibition of cellular entry of lymphocytic choriomeningitis virus by amphipathic DNA polymers Virology 2008 372 107 117 10.1016/j.virol.2007.10.016 18022208 PMC2821746

[B8] Matsumura T Hu Z Kato T Dreux M Zhang YY Imamura M Hiraga N Juteau JM Cosset FL Chayama K Amphipathic DNA polymers inhibit hepatitis C virus infection by blocking viral entry Gastroenterology 2009 137 673 681 10.1053/j.gastro.2009.04.048 19394333 PMC2803092

[B9] Weissenhorn W Dessen A Harrison SC Skehel JJ Wiley DC Atomic structure of the ectodomain from HIV-1 gp41 Nature 1997 387 426 430 10.1038/387426a0 9163431

[B10] Gianni T Martelli PL Casadio R Campadelli-Fiume G The ectodomain of herpes simplex virus glycoprotein H contains a membrane alpha-helix with attributes of an internal fusion peptide, positionally conserved in the herpesviridae family J Virol 2005 79 2931 2940 10.1128/JVI.79.5.2931-2940.2005 15709012 PMC548475

[B11] Heldwein EE Lou H Bender FC Cohen GH Eisenberg RJ Harrison SC Crystal structure of glycoprotein B from herpes simplex virus 1 Science 2006 313 217 220 10.1126/science.1126548 16840698

[B12] Eschli B Quirin K Wepf A Weber J Zinkernagel R Hengartner H Identification of an N-terminal trimeric coiled-coil core within arenavirus glycoprotein 2 permits assignment to class I viral fusion proteins J Virol 2006 80 5897 5907 10.1128/JVI.00008-06 16731928 PMC1472595

[B13] Wilson IA Skehel JJ Wiley DC Structure of the haemagglutinin membrane glycoprotein of influenza virus at 3 A resolution Nature 1981 289 366 373 10.1038/289366a0 7464906

[B14] Zhao X Singh M Malashkevich VN Kim PS Structural characterization of the human respiratory syncytial virus fusion protein core Proc Natl Acad Sci USA 2000 97 14172 14177 10.1073/pnas.260499197 11106388 PMC18890

[B15] Dimitrov DS Virus entry: molecular mechanisms and biomedical applications Nat Rev Microbiol 2004 2 109 122 10.1038/nrmicro817 15043007 PMC7097642

[B16] de Smet MD Meenken CJ Horn GJ van den Fomivirsen - a phosphorothioate oligonucleotide for the treatment of CMV retinitis Ocul Immunol Inflamm 1999 7 189 198 10.1076/ocii.7.3.189.4007 10611727

[B17] Bernstein DI Goyette N Cardin R Kern ER Boivin G Ireland J Juteau JM Vaillant A Amphipathic DNA polymers exhibit antiherpetic activity in vitro and in vivo Antimicrob Agents Chemother 2008 52 2727 2733 10.1128/AAC.00279-08 18505857 PMC2493138

[B18] Krieg AM Efler SM Wittpoth M Al Adhami MJ Davis HL Induction of systemic TH1-like innate immunity in normal volunteers following subcutaneous but not intravenous administration of CPG a synthetic B-class CpG oligodeoxynucleotide TLR9 agonist J Immunother 7909 27 460 471 10.1097/00002371-200411000-00006 15534490

[B19] MacDonald MR Li XY Stenberg RM Campbell AE Virgin HWt Mucosal and parenteral vaccination against acute and latent murine cytomegalovirus (MCMV) infection by using an attenuated MCMV mutant J Virol 1998 72 442 451 9420244 10.1128/jvi.72.1.442-451.1998 PMC109393

[B20] Cardin RD Brooks JW Sarawar SR Doherty PC Progressive loss of CD8+ T cell-mediated control of a gamma-herpesvirus in the absence of CD4+ T cells J Exp Med 1996 184 863 871 10.1084/jem.184.3.863 9064346 PMC2192775

[B21] Cardin RD Schaefer GC Allen JR Davis-Poynter NJ Farrell HE The M33 chemokine receptor homolog of murine cytomegalovirus exhibits a differential tissue-specific role during in vivo replication and latency J Virol 2009 83 7590 7601 10.1128/JVI.00386-09 19439478 PMC2708650

[B22] Bravo FJ Cardin RD Bernstein DI Effect of maternal treatment with cyclic HPMPC in the guinea pig model of congenital cytomegalovirus infection J Infect Dis 2006 193 591 597 10.1086/499603 16425139

[B23] Wagner M Michel D Schaarschmidt P Vaida B Jonjic S Messerle M Mertens T Koszinowski U Comparison between human cytomegalovirus pUL97 and murine cytomegalovirus (MCMV) pM97 expressed by MCMV and vaccinia virus: pM97 does not confer ganciclovir sensitivity J Virol 2000 74 10729 10736 10.1128/JVI.74.22.10729-10736.2000 11044117 PMC110947

[B24] Bale JF Jr O'Neil ME Detection of murine cytomegalovirus DNA in circulating leukocytes harvested during acute infection of mice J Virol 1989 63 2667 2673 2542580 10.1128/jvi.63.6.2667-2673.1989 PMC250753

[B25] Cardin RD Boname JM Abenes GB Jennings SA Mocarski ES Plotkin SA, Michelson S Reactivation of murine cytomegalovirus from latency Multidisciplinary Approaches to Understanding Cytomegalovirus Disease 1993 Amsterdam: Elsevier 101 110

[B26] Collins TM Quirk MR Jordan MC Biphasic viremia and viral gene expression in leukocytes during acute cytomegalovirus infection of mice J Virol 1994 68 6305 6311 8083970 10.1128/jvi.68.10.6305-6311.1994 PMC237051

[B27] Hudson JB The murine cytomegalovirus as a model for the study of viral pathogenesis and persistent infections Arch Virol 1979 62 1 29 10.1007/BF01314900 231945

[B28] Stoddart CA Cardin RD Boname JM Manning WC Abenes GB Mocarski ES Peripheral blood mononuclear phagocytes mediate dissemination of murine cytomegalovirus J Virol 1994 68 6243 6253 8083964 10.1128/jvi.68.10.6243-6253.1994 PMC237044

[B29] Agrawal S Temsamani J Tang JY Pharmacokinetics, biodistribution, and stability of oligodeoxynucleotide phosphorothioates in mice Proc Natl Acad Sci USA 1991 88 7595 7599 10.1073/pnas.88.17.7595 1881900 PMC52348

[B30] Saijo Y Perlaky L Wang H Busch H Pharmacokinetics, tissue distribution, and stability of antisense oligodeoxynucleotide phosphorothioate ISIS 3466 in mice Oncol Res 1994 6 243 249 7865900

[B31] Geary RS Leeds JM Henry SP Monteith DK Levin AA Antisense oligonucleotide inhibitors for the treatment of cancer: 1. Pharmacokinetic properties of phosphorothioate oligodeoxynucleotides Anticancer Drug Des 1997 12 383 393 9236854

[B32] Phillips JA Craig SJ Bayley D Christian RA Geary R Nicklin PL Pharmacokinetics, metabolism, and elimination of a 20-mer phosphorothioate oligodeoxynucleotide (CGP 69846A) after intravenous and subcutaneous administration Biochem Pharmacol 1997 54 657 668 10.1016/S0006-2952(97)00190-1 9310342

[B33] Yu RZ Geary RS Leeds JM Watanabe T Moore M Fitchett J Matson J Burckin T Templin MV Levin AA Comparison of pharmacokinetics and tissue disposition of an antisense phosphorothioate oligonucleotide targeting human Ha-ras mRNA in mouse and monkey J Pharm Sci 2001 90 182 193 10.1002/1520-6017(200102)90:2<182::AID-JPS9>3.0.CO;2-F 11169535

[B34] Yu RZ Kim TW Hong A Watanabe TA Gaus HJ Geary RS Cross-species pharmacokinetic comparison from mouse to man of a second-generation antisense oligonucleotide, ISIS 30 targeting human apolipoprotein B-100 Drug Metab Dispos 1012 35 460 468 10.1124/dmd.106.012401 17172312

[B35] Yang T Arnold JJ Ahsan F Tetradecylmaltoside (TDM) enhances in vitro and in vivo intestinal absorption of enoxaparin, a low molecular weight heparin J Drug Target 2005 13 29 38 10.1080/10611860400020191 15848952

[B36] Maggio ET Intravail: highly effective intranasal delivery of peptide and protein drugs Expert Opin Drug Deliv 2006 3 529 539 10.1517/17425247.3.4.529 16822227

[B37] Raoof AA Chiu P Ramtoola Z Cumming IK Teng C Weinbach SP Hardee GE Levin AA Geary RS Oral bioavailability and multiple dose tolerability of an antisense oligonucleotide tablet formulated with sodium caprate J Pharm Sci 2004 93 1431 1439 10.1002/jps.20051 15124202

[B38] Tillman LG Geary RS Hardee GE Oral delivery of antisense oligonucleotides in man J Pharm Sci 2008 97 225 236 10.1002/jps.21084 17721945

[B39] Crooke RM Graham MJ Lemonidis KM Whipple CP Koo S Perera RJ An apolipoprotein B antisense oligonucleotide lowers LDL cholesterol in hyperlipidemic mice without causing hepatic steatosis J Lipid Res 2005 46 872 884 10.1194/jlr.M400492-JLR200 15716585

[B40] Kastelein JJ Wedel MK Baker BF Su J Bradley JD Yu RZ Chuang E Graham MJ Crooke RM Potent reduction of apolipoprotein B and low-density lipoprotein cholesterol by short-term administration of an antisense inhibitor of apolipoprotein B Circulation 2006 114 1729 1735 10.1161/CIRCULATIONAHA.105.606442 17030687

[B41] Gianni T Menotti L Campadelli-Fiume G A heptad repeat in herpes simplex virus 1 gH, located downstream of the alpha-helix with attributes of a fusion peptide, is critical for virus entry and fusion J Virol 2005 79 7042 7049 10.1128/JVI.79.11.7042-7049.2005 15890943 PMC1112143

[B42] Olbrich AR Schimmer S Heeg K Schepers K Schumacher TN Dittmer U Effective postexposure treatment of retrovirus-induced disease with immunostimulatory DNA containing CpG motifs J Virol 2002 76 11397 11404 10.1128/JVI.76.22.11397-11404.2002 12388700 PMC136771

[B43] Dong L Mori I Hossain MJ Liu B Kimura Y An immunostimulatory oligodeoxynucleotide containing a cytidine-guanosine motif protects senescence-accelerated mice from lethal influenza virus by augmenting the T helper type 1 response J Gen Virol 2003 84 1623 1628 10.1099/vir.0.19029-0 12771433

[B44] Harandi AM Eriksson K Holmgren J A protective role of locally administered immunostimulatory CpG oligodeoxynucleotide in a mouse model of genital herpes infection J Virol 2003 77 953 962 10.1128/JVI.77.2.953-962.2003 12502811 PMC140825

[B45] Compton T Kurt-Jones EA Boehme KW Belko J Latz E Golenbock DT Finberg RW Human cytomegalovirus activates inflammatory cytokine responses via CD14 and Toll-like receptor 2 J Virol 2003 77 4588 4596 10.1128/JVI.77.8.4588-4596.2003 12663765 PMC152130

[B46] Krug A French AR Barchet W Fischer JA Dzionek A Pingel JT Orihuela MM Akira S Yokoyama WM Colonna M TLR9-dependent recognition of MCMV by IPC and DC generates coordinated cytokine responses that activate antiviral NK cell function Immunity 2004 21 107 119 10.1016/j.immuni.2004.06.007 15345224

[B47] Tabeta K Georgel P Janssen E Du X Hoebe K Crozat K Mudd S Shamel L Sovath S Goode J Toll-like receptors 9 and 3 as essential components of innate immune defense against mouse cytomegalovirus infection Proc Natl Acad Sci USA 2004 101 3516 3521 10.1073/pnas.0400525101 14993594 PMC373494

[B48] Szomolanyi-Tsuda E Liang X Welsh RM Kurt-Jones EA Finberg RW Role for TLR2 in NK cell-mediated control of murine cytomegalovirus in vivo J Virol 2006 80 4286 4291 10.1128/JVI.80.9.4286-4291.2006 16611887 PMC1472014

[B49] Tian P Shen X Feng H Gao B IL-1B Attenuates IFN-aB-induced antiviral activity and STAT1 activation in the liver: involvement of proteasome-dependent pathway J Immunol 2000 165 3959 3965 11034404 10.4049/jimmunol.165.7.3959

[B50] Anderson KP Lie YS Low MA Fennie EH Effects of tumor necrosis factor-alpha treatment on mortality in murine cytomegalovirus-infected mice Antiviral Res 1993 21 343 355 10.1016/0166-3542(93)90012-8 8215304

[B51] Kanehara H Mizuguchi M Tajima K Kanaori K Makino K Spectroscopic evidence for the formation of four-stranded solution structure of oligodeoxycytidine phosphorothioate Biochemistry 1997 36 1790 1797 10.1021/bi961528c 9048563

[B52] Kern ER Pivotal role of animal models in the development of new therapies for cytomegalovirus infections Antiviral Res 2006 71 164 171 10.1016/j.antiviral.2006.05.018 16828175

